# The running suture with clip and string technique after colonic endoscopic submucosal dissection

**DOI:** 10.1055/a-2271-6925

**Published:** 2024-04-18

**Authors:** Mai Ego Makiguchi, Yutaka Saito

**Affiliations:** 1 68380 Endoscopy Division, National Cancer Center Hospital, Chuo-ku, Tokyo, Japan

A closure technique to prevent bleeding after colorectal endoscopic submucosal dissection (ESD) has previously been reported; Nishizawa et al. reported a string and clip closure technique using the following method 1 . The clip and string can be passed through the instrument channel of a single-channel endoscope and is placed at the distal edge of a large mucosal defect. A second clip is hooked onto the string and placed on the opposite side. Both clips are gathered by pulling the free end of the string, and additional clips are placed to achieve complete closure. This technique has limitations however on the size of the defect that can be completely closed. Therefore, we adapted this method and developed a new closure technique for colorectal ESD ( Video 1 ).

A running suture is created using a new clip and string technique after colorectal ESD.Video 1

A 70-year-old woman was diagnosed with a 30-mm lesion in the cecum ( Fig. 1 **a** ). After ESD had been performed ( Fig. 1 **b** ), the scope was not removed and a clip with string was inserted through the instrument channel. The clip with its attached string was placed on the distal edge of the mucosal defect, and the string was then pulled from the instrument channel. A second clip was placed over the string on the proximal edge of the mucosal defect and the string was used to pull together the proximal and distal sides of the mucosal defect. Subsequently, the string was hooked under a third clip, with this and the subsequent clips being placed on the non-tumorous mucosa alternately on the distal and proximal sides, hooking the string each time ( Fig. 1 **c** ). Finally, the string outside the channel was pulled tight to close the mucosal defect ( Fig. 1 **d** ) and was then cut with scissors forceps (Olympus, Japan).

**Fig. 1:**
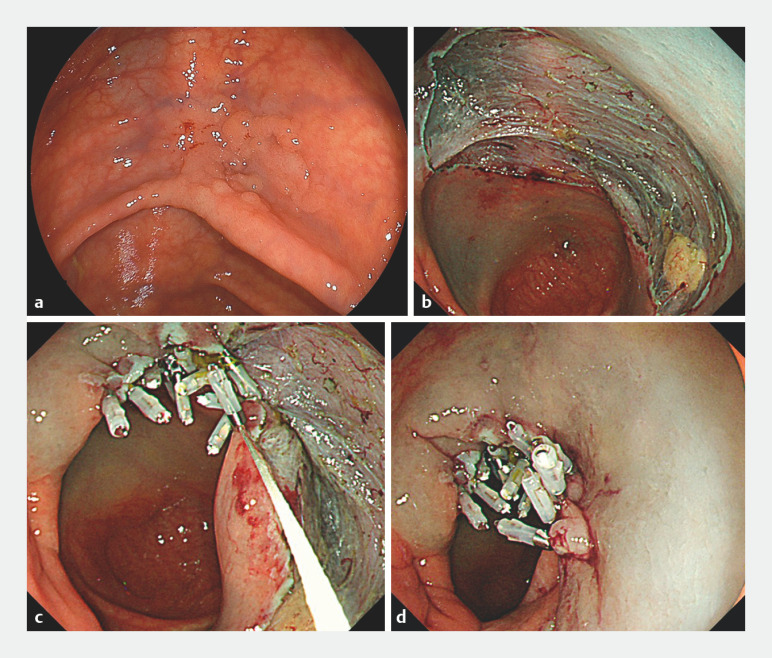
**a** Endoscopic images showing: a a 30-mm flat elevated lesion in the cecum; **b** the mucosal defect left after endoscopic submucosal dissection; **c** clips placed on the nontumorous mucosa alternately on the distal and proximal sides; **d** complete closure of the mucosal defect with the consecutive closure method.

A similar method, the reopenable clip-over-the-line method (ROLM), has also been reported 2 , but requires the use of a Sureclip (MC Medical. Japan), while our method can use reloadable clips, which are characterized by their low cost. This method is similar to ROLM, but by using Eco Easy-Clip, it can be named EOLM (Easy & Eco clip over the Line Method). This is a simple technique, but a prospective study is required to confirm its efficacy and safety. In conclusion, a running suture with the clip and string technique appears to be feasible.

Endoscopy_UCTN_Code_TTT_1AQ_2AD

## Notes

Endoscopy E-Videos https://eref.thieme.de/e-videos
*E-Videos* is an open access online section of the journal *Endoscopy* , reporting on interesting cases and new techniques in gastroenterological endoscopy. All papers include a high-quality video and are published with a Creative Commons CC-BY license. Endoscopy E-Videos qualify for HINARI discounts and waivers and eligibility is automatically checked during the submission process. We grant 100% waivers to articles whose corresponding authors are based in Group A countries and 50% waivers to those who are based in Group B countries as classified by Research4Life (see: https://www.research4life.org/access/eligibility/ ). This section has its own submission website at https://mc.manuscriptcentral.com/e-videos .
